# Low transferrin saturation (TSAT) and high ferritin levels are significant predictors for cerebrovascular and cardiovascular disease and death in maintenance hemodialysis patients

**DOI:** 10.1371/journal.pone.0236277

**Published:** 2020-09-02

**Authors:** Takahiro Kuragano, Nobuhiko Joki, Hiroki Hase, Kenichiro Kitamura, Toshiaki Murata, Shouichi Fujimoto, Atushi Fukatsu, Toru Inoue, Yukihiro Itakura, Takeshi Nakanishi

**Affiliations:** 1 Department of Internal Medicine, Division of Kidney and Dialysis, Hyogo College of Medicine, Nisinomiya, Japan; 2 Department of Nephrology, Toho University Ohashi Medical Center, Tokyo, Japan; 3 The Third Department of Internal Medicine Faculty of Medicine, The University of Yamanashi, Yamanashi, Japan; 4 Department of Nephrology, Murakami karin dou Hospital, Fukuoka, Japan; 5 Department of Hemovascular Medicine and Artificial Organs, Faculty of Medicine, University of Miyazaki, Miyazaki, Japan; 6 Department of Nephrology, Hekinan Municipal Hospital, Hekinan, Japan; 7 Department of Internal Medicine, Yuseikai Clinic, Osaka, Japan; 8 Department of Internal Medicine, Itakura Clinic, Hekinan, Japan; Medizinische Fakultat der RWTH Aachen, GERMANY

## Abstract

Patients with high serum ferritin and low transferrin saturation (TSAT) levels could be considered as presenting with dysutilization of iron for erythropoiesis. However, the long-term safety of iron administration in these patients has not been well established. An observational multicenter study was performed over 3 years. In 805 patients undergoing maintenance hemodialysis (MHD), we defined dysutilization of iron for erythropoiesis in patients with lower TSAT (<20%) and higher ferritin (≥100 ng/mL) levels. A time-dependent Cox hazard model was used for the evaluation of the association between dysutilization of iron for erythropoiesis and adverse events and survival. Patients with low TSAT levels showed an increased risk of cerebrovascular and cardiovascular disease (CCVD) and death compared to patients with normal or higher TSAT levels. Patients with low ferritin and high TSAT levels had a significantly lower risk of CCVD and death compared with patients with high ferritin and low TSAT levels. Higher TSAT levels were associated with male gender, age, the absence of diabetes, low levels of high-sensitivity CRP, and low β2 microglobulin levels, but not with intravenous iron administration or ferritin levels. Although patients with low TSAT levels had a significantly higher risk of CCVD or death, high TSAT levels were not linked with iron administration. Patients, who were suspected of dysutilization of iron for erythropoiesis, had a higher risk of CCVD and death. The administration of iron should be performed cautiously for improving TSAT levels, as iron administration could sustain TSAT levels for a short term.

## Introduction

Currently, several international guidelines [1,2] regarding the treatment of anemia with chronic kidney disease have recommended ferritin and TSAT as indexes of iron status, as well as markers for the initiation and cessation of iron supplementation in CKD patients. The serum ferritin level is a widely used peripheral iron biomarker. It is thought to be correlated with iron stores in the absence of inflammation, while serum ferritin levels are reflected by the iron storage with the amplification of inflammation. Then, low serum ferritin levels generally indicate absolute? iron deficiency accurately. Transferrin saturation (TSAT), the ratio of serum iron to total iron-binding capacity, is also considered an important biochemical marker of overall bodily iron status, which can be used to monitor response to ESA (erythropoiesis-stimulating agent) and/or iron therapy in CKD [3]. Despite its valuable clinical utility, several limitations can affect the ability of TSAT to accurately reflect body iron states [3]. Serum iron levels fluctuate diurnally and can change acutely depending on dietary iron intake. Furthermore, in the case of inflammation, serum iron levels rapidly decrease by the sequestration of iron in macrophages. Thus, low TSAT levels can reflect not only iron deficiency but also inflammatory and nutritional conditions. Both low ferritin and low TSAT levels reflect an absolute iron deficiency. On the other hand, patients with high ferritin and TSAT levels were suspected of dysutilization of iron for erythropoiesis. The dysutilization of iron for erythropoiesis is a state in which there is insufficient iron incorporation into erythroid precursors in the face of apparently adequate body iron stores [4]. This condition is seen in patients with infectious diseases, chronic inflammation, chronic heart disease, chronic kidney disease, and malignant disease [5,6]. The Dialysis Patients Response to IV Iron with Elevated Ferritin (DRIVE) study reported the efficacy of intravenous iron administration in the improvement of Hb levels in MHD patients who had ferritin levels of 500–1200 ng/mL and TSAT levels under 25%, i.e., in those who were suspected of dysutilization of iron for erythropoiesis [7]. Moreover, a recent meta-analysis based on 34 studies involving a total of 2,658 MHD patients demonstrated that administration of intravenous iron to patients with serum ferritin levels >200 ng/mL with or without a TSAT <30% could effectively improve anemia in these patients [8]. From the results of these studies, it is possible that iron administration in patients with dysutilization of iron might increase Hb levels, even though they store excess iron. However, the long-term effectiveness and safety of continuous iron administration in these patients were not confirmed in these studies. Therefore, in this study, we investigated the potential role of TSAT levels, as well as the relationship between the dysutilization of iron for erythropoiesis and adverse events and/or survival, in maintenance hemodialysis (MHD) patients.

## Materials and methods

### Study design

In the present study, we reanalyzed data from the prospective treatment for renal anemia on prognosis in hemodialysis patients (TRAP) study, which revealed an association between ferritin or Hb level fluctuations and adverse events in MHD patients. The design and methods of the TRAP study have been previously reported [9]. Briefly, the TRAP study design was a prospective, multicenter observational study. The duration of the study was three years, which was performed in Japan since June 2007. The anticipated trial start date was April 2007, and Last follow-up date was April 2014.

### Patients

**Fig 1:**
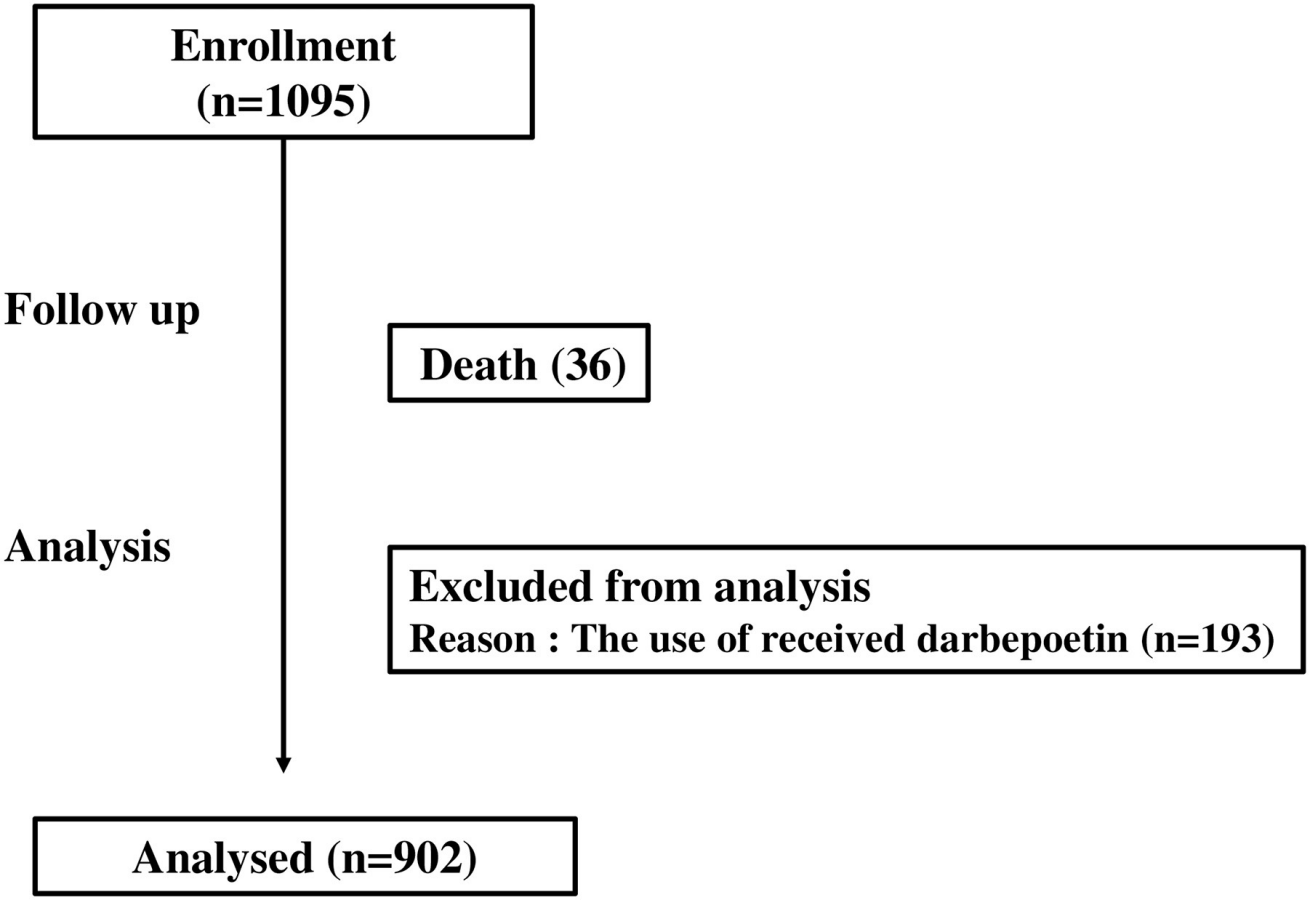
Flow diagram of the progress of this study.

Patients who were on MHD were recruited for this study. Patients who had received MHD for <1 year, patients older than 75 years, patients with chronic inflammation, malignancy, hematological disorders, or severe liver dysfunction, and patients who had received anti-inflammatory drugs or immunosuppressive agents were excluded from this study (Fig 1). The protocol was approved in accordance with the ethical principles outlined in the 1975 Declaration of Helsinki as revised in 2013 by the Ethics Review Board of the Hyogo College of Medicine (approval number 419). Written informed consent was obtained from all patients. The study was registered with the University Hospital Medical Information Network (UMIN) Clinical Trial Registry (UMIN000000687).

### Measurements

The blood levels of Hb, ferritin, iron, total iron binding capacity (TIBC), β2-microglobulin (β2-MG), creatinine (Cr), total protein (TP), albumin, total cholesterol (T-CHO), low-density lipoprotein cholesterol (LDL-CHO), triglycerides (TG), calcium, and phosphate were measured every 3 months. Doses of erythropoiesis-stimulating agents (ESA) and iron were evaluated during the study period. The blood levels of high-sensitivity C-reactive protein (CRP) and intact-parathyroid hormone (int-PTH) were also measured every six months. TSAT was calculated with the following formula: iron / TIBC x 100.

### TSAT fluctuation patterns

Patients were divided into the following 6 categories based on TSAT fluctuation patterns: (1) Target TSAT group: within the target TSAT range (20–30%), (2) Low TSAT group: consistently below the target TSAT range, (3) High TSAT group: consistently above the target TSAT range, (4) LAH-TSAT group: low-amplitude fluctuation around the upper limit of the target TSAT range (30%), (5) LAL-TSAT group: low-amplitude fluctuation around the lower limit of the target TSAT range (20%), and (6) HA-TSAT group: high-amplitude fluctuation across the target-TSAT range.

### Ferritin and TSAT categories

The 2006 Kidney Disease Outcome Quality Initiative (KDOQI) proposed criteria for absolute iron deficiency that included patients with ferritin levels under 100 ng/mL and TSAT levels under 20% [10]. Thus, we divided patients into the following 4 groups according to serum ferritin and TSAT levels: (1) low ferritin (<100 ng/mL) and low TSAT (<20%) group, (2) low ferritin and high TSAT (≥20%) group, (3) high ferritin (≥100 ng/mL) and low TSAT group, and (4) high ferritin and high TSAT group.

### Definition of adverse events

Adverse events were diagnosed by physicians in each facility. Congestive heart failure and ischemic heart disease (e.g., angina or acute myocardial infarction) were defined as cardiovascular disease, and cerebral infarction and cerebral hemorrhage were defined as cerebrovascular disease. Cardiovascular disease was diagnosed by electrocardiogram, blood biochemistry testing or cardiac ultrasound. Cerebral infarction or cerebral hemorrhage were diagnosed by computer tomography or magnetic resonance imaging.

### Analysis

#### The determinants of TSAT and the relationship between fluctuation patterns of TSAT and adverse events

To analyze the determinants of higher TSAT levels, we utilized stepwise multiple linear regression analysis. Furthermore, to evaluate the relationship between variance patterns of TSAT and adverse events, a time-dependent proportional hazard model was employed that used each event as the dependent variable and the fluctuation patterns of TSAT as independent variables. We classified the variation patterns of TSAT using the preceding three years of data as the evaluation point for each patient, which were defined as covariates because these factors change with time. To analyze the correlation between fluctuation patterns of TSAT and adverse events, forward selection was used to choose covariates from the following adjustment factors: age, sex, time on HD treatment, with or without DM (diabetes mellitus), with or without CVD, levels of creatinine, β2-MG, ferritin, Hb, BMI (body mass index), hCRP, albumin, int-PTH, and dosage of ESA.

#### Comparison of clinical parameters among various TSAT levels

We compared serum β2-MG, hCRP, Cr, and albumin levels among TSAT groups (<20, 20–29, 30–39, 40 ≤) by mixed-model multiple pairwise comparisons test with Bonferroni correction. We further compared TSAT levels between groups with or without iron administration. We compared TSAT levels between patients with or without intravenous iron administration past three months by mixed-model multiple pairwise comparisons test with Bonferroni correction.

#### The relationship between ferritin levels, TSAT categories and adverse events

To evaluate the relationship between ferritin levels, TSAT categories and adverse events, a time-dependent proportional hazard model was also employed using each event as the dependent variable, and the ferritin levels and TSAT categories as independent variables. The data were adjusted based on age, sex, time on HD treatment, presence or absence of DM, presence or absence of CVD, levels of albumin, creatinine, β2-MG, BMI, int-PTH, Hb, hCRP, and dosage of ESA.

Statistical analyses were performed using SPSS version 18.0 software (IBM, Inc., Chicago, IL, USA) and R ver. 2.13.0 (R Core Team (2011). R is a language and operating environment for statistical computing available from the R Foundation for Statistical Computing, Vienna, Austria. URL http://www.R-project.org/), and MedCalc ver. 12.7.7 (MedCalc Software, Ostend, Belgium) was also used for analysis.

## Results

### Baseline characteristics of patients

The mean age of the study patients was 61.8±9.9 years old, and 60.3% were male. The percentage of patients with DM was 33.7%, the mean time of dialysis was 106±101 months, and the mean dose of ESA was 3212 ±2107 (IU/Week). The percentage of patients treated with intravenous iron was 20.8%. The mean levels of Hb, ferritin, TSAT, albumin, and int-PTH were 10.6±1.0 g/dL, 78.5±48.3 ng/mL, 26.7±11.7%, 3.7±0.3 g/dL, and 124 ±88.6 pg/mL, respectively. All patients were treated with epoetin α or β. ESA or IV iron administration was performed according to the 2008 Japanese Society for Dialysis Therapy: Guidelines for Renal Anemia in Chronic Kidney Disease [11].

### The relationship between TSAT levels and adverse events in MHD patients

**Fig 2:**
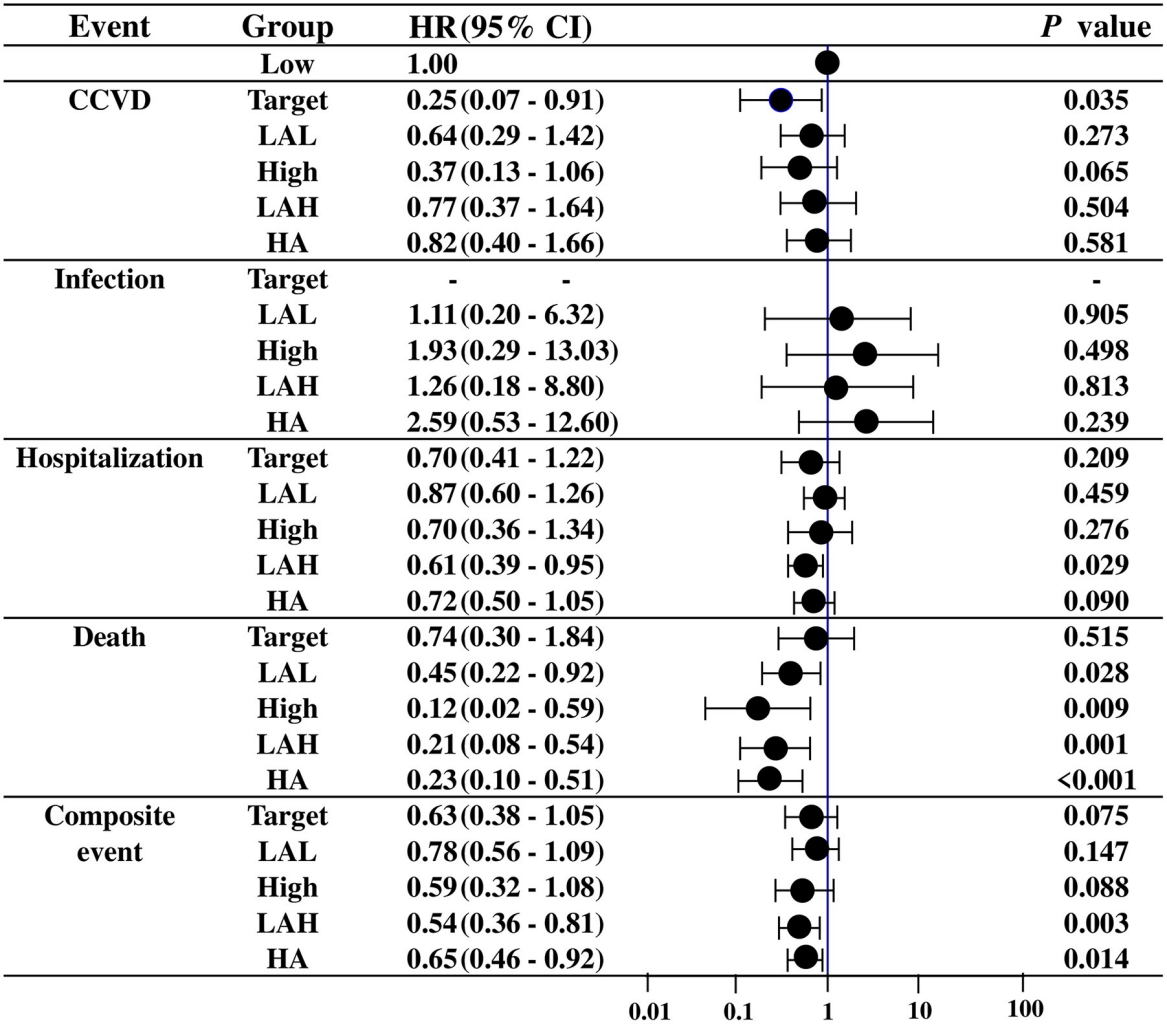
The relationship between TSAT levels and adverse events in MHD patients. Target: within the target TSAT range (20–30%). L: Low: consistently below the target TSAT range. LAL: Low-amplitude fluctuation around the lower limit of the target TSAT range. H, High: consistently above the target TSAT range. LAH, LAH-TSAT group: low-amplitude fluctuation around the upper limit of the target TSAT range. H, HA: high-amplitude fluctuation across the target-TSAT range. CCVD: cerebrovascular and cardiovascular disease. Composite events: CCVD, infection, hospitalization, and death.

In a time-dependent Cox hazard model, compared to those with low (≤ 20%) TSAT levels, patients with target (20–30%) TSAT levels had significantly lower risks for cerebrovascular and cardiovascular disease (CCVD) (HR: 0.25, P = 0.035), and patients with higher (≥30%) TSAT level had a significantly lower risk of death (HR: 0.12, P = 0.009) (Fig 2).

### Predictors of high TSAT levels in MHD patients

**Fig 3:**
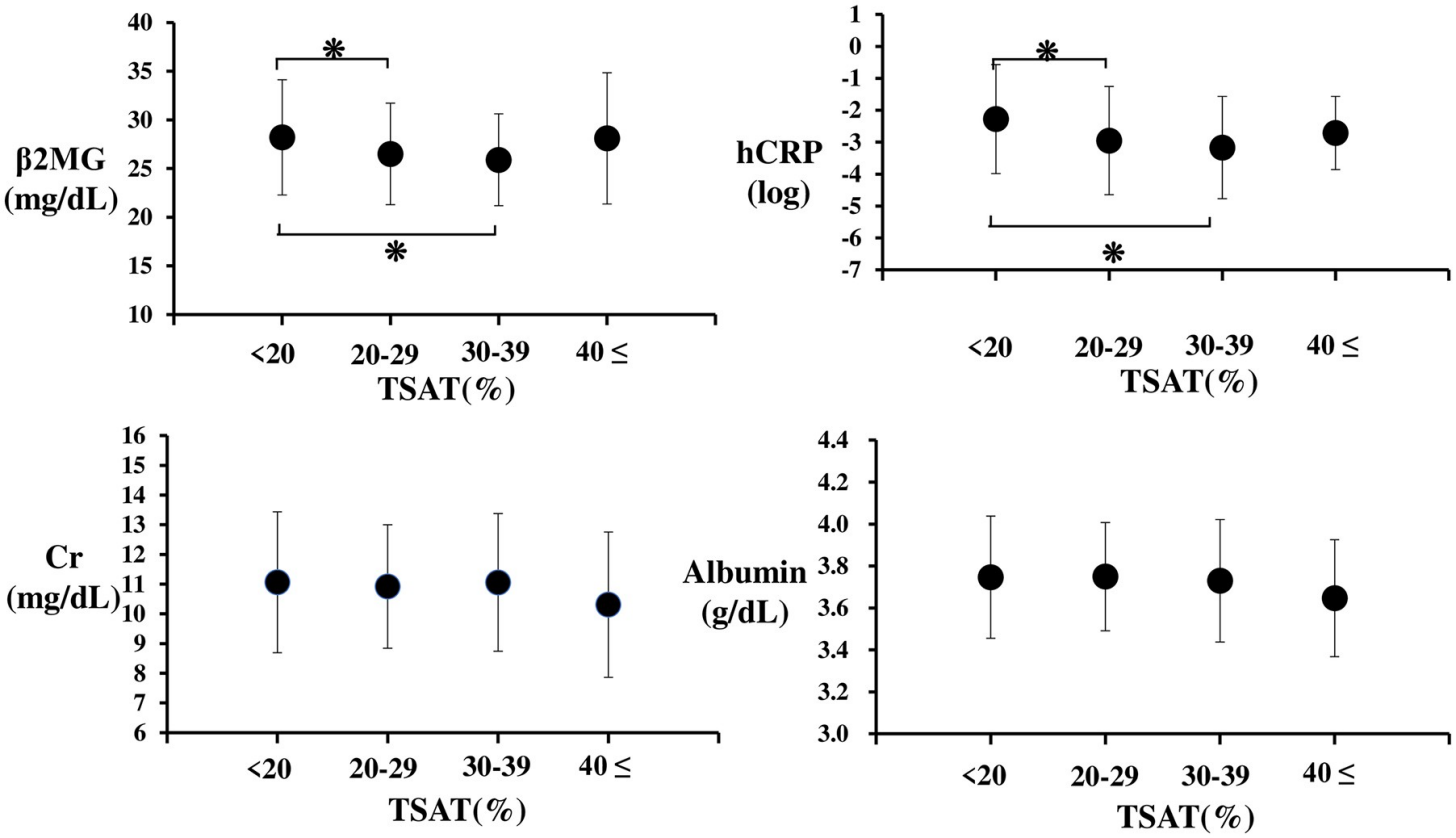
Comparison of clinical parameters among patients with various TSAT levels. There was no significant difference in the serum Cr and albumin levels among TSAT groups. On the other hand, serum β2-MG and hCRP levels in patients with TSAT levels under 20% were significantly higher than in patients with TSAT levels of 20–29 and 30–39%. ❋: p<0.05, Data represent means ± SD.

**Fig 4:**
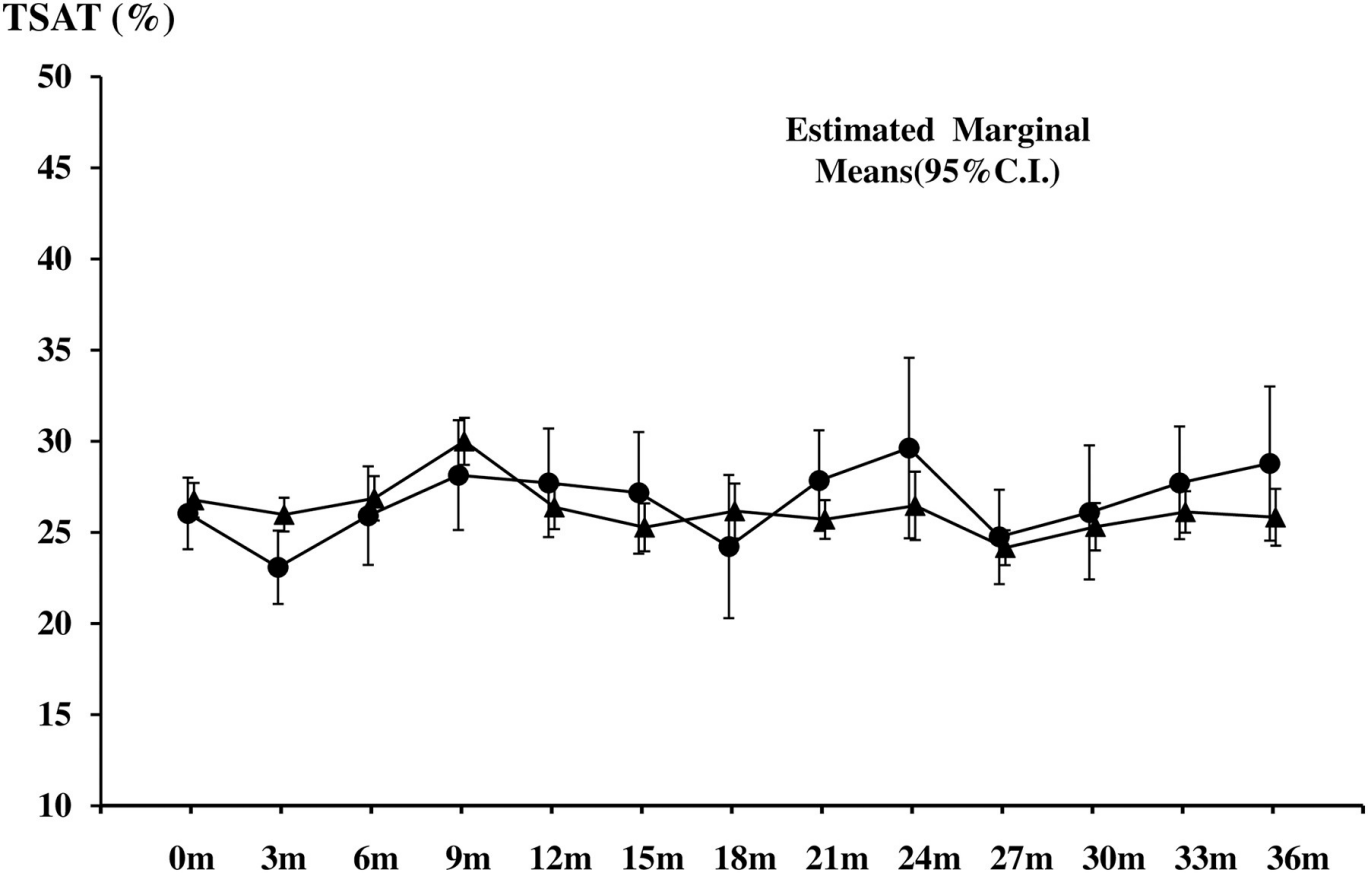
Comparison of TSAT levels between patients receiving or not receiving intravenous iron administration. During the observational period, there was no significant difference in TSAT levels between patients treated with intravenous iron in the past three months and those without this treatment. ●: patients not treated with intravenous iron, ▲: patients treated with intravenous iron. Data represent means ± SD.

In multivariate logistic regression analysis, male patients (β-coefficient 0.567, P = 0.028), younger patients (β-coefficient 0.036, P = 0.011), patients without diabetes (β-coefficient 0.748, P = 0.02), patients with low hCRP levels (β-coefficient 0.46, P = 0.001), and patients with low β2-MG (β-coefficient 0.666, P = 0.001) levels were identified as significant predictors of high TSAT levels, although intravenous iron administration or ferritin levels were not (Table 1). Patients were divided into 4 groups according to their TSAT level (<20, 20–29, 30–39, 40%≤) at the start of the study. Serum β2-MG and hCRP levels in patients with TSAT levels of 20–39% were significantly lower than those in patients with TSAT levels <20%. There was no significant difference in the serum Cr and albumin levels among the 4 groups (Fig 3). Furthermore, there was no significant difference in TSAT levels between patients treated with intravenous iron (in the past 3 months) and patients without intravenous iron administration during the study period (Fig 4).

**Table 1:** Predictors for higher TSAT (≥20%) levels in MHD patients (stepwise multiple linear regression analysis).

Independent variables	β-coefficient	SEM	P-value
**Female**	**-0.567**	**0.257**	**0.028**
**Age**	**-0.036**	**0.14**	**0.011**
**DM**	**-0.748**	**0.246**	**0.02**
**hCRP**	**-0.46**	**0.091**	**0.001**
**β2MG**	**-0.066**	**0.02**	**0.001**

Dependent variables: Sex, age, duration of dialysis, DM, CVD · Hb · ferritin · BMI · albumin · hCRP · int-PTH · creatinine · β2MG · Dose of ESA · Dose of intravenous iron.

### The relationship between ferritin or TSAT categories and adverse events in MHD patients

**Fig 5:**
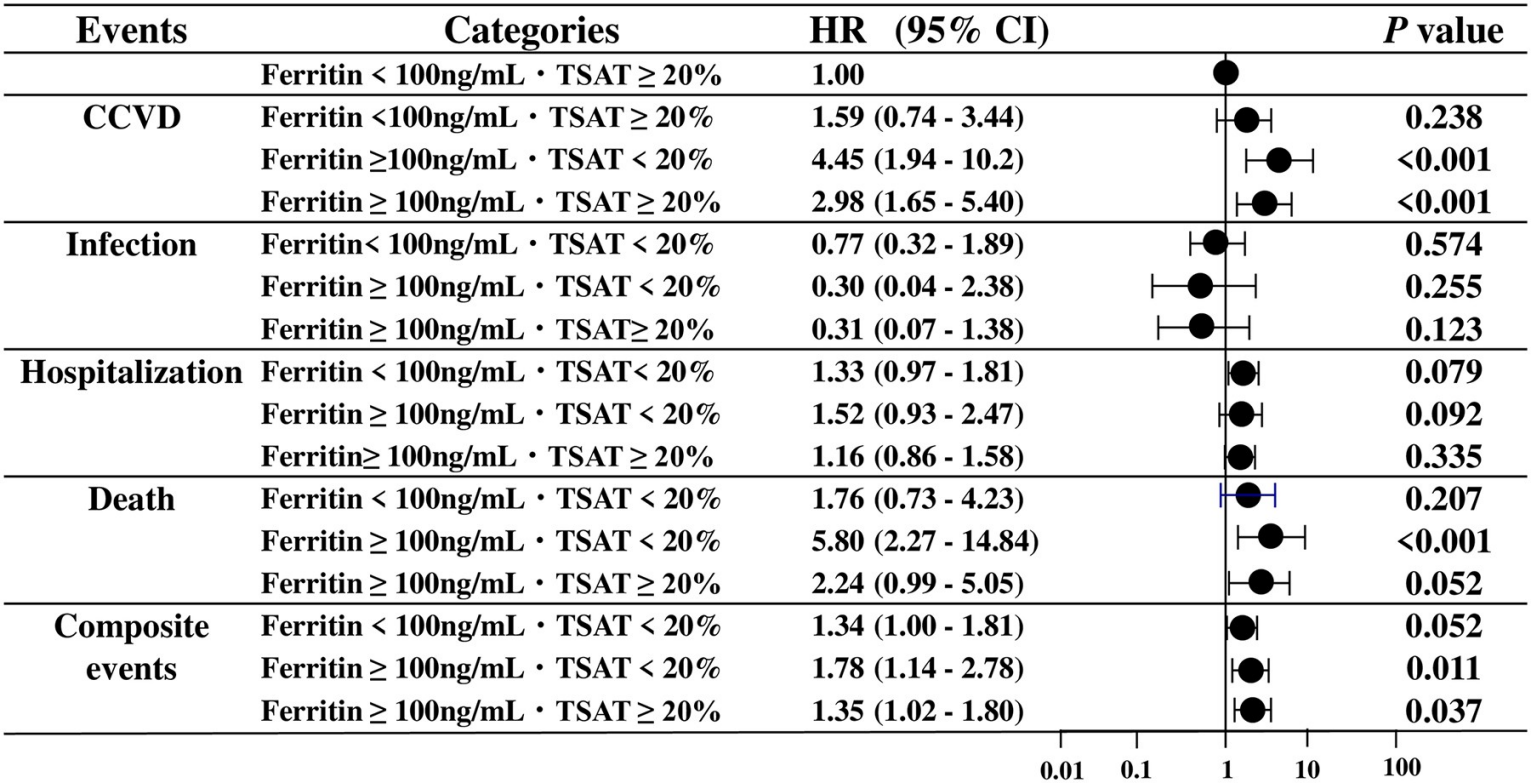
The relationship between ferritin levels, TSAT categories, and adverse events. CCVD: cerebrovascular and cardiovascular disease. Composite events: CCVD, infection, hospitalization, and death.

Compared with low ferritin (<100 ng/mL) and high TSAT (≥20%) patients, patients with high ferritin (≥100 ng/mL) and low TSAT (<20%) (HR: 4.45, p<0.001) and with high ferritin (≥100 ng/mL) and high TSAT (≥20%) (HR: 2.98, p<0.001) had a significantly higher risk of CCVD. Patients with high ferritin levels (≥100 ng/mL) and low TSAT (<20%) had a significantly higher risk of death (HR: 5.8, p<0.001) compared to patients with low ferritin and high TSAT. Moreover, patients with low ferritin and high TSAT had a significantly lower risk for composite events than patients in the high ferritin and low TSAT group (HR: 1.78, p = 0.011) and the high ferritin and high TSAT group (HR: 1.35, p = 0.037) (Fig 5).

## Discussion

### TSAT and adverse events in MHD patients

It is well-established that serum iron levels can fluctuate dramatically depending on dietary iron intake or patient condition. Serum iron has further been identified as a variable causing fluctuations of TSAT. Therefore, to investigate the relationship between TSAT and adverse events in MHD patients, an evaluation that considered the fluctuation patterns was needed. In this study, we found that, in comparison to patients with low TSAT (≤ 20%) values, patients with target TSAT (20–30%) levels were at a significantly lower risk of developing CCVD, and patients with higher TSAT (≥30%) levels further had a significantly lower risk of death. Moreover, patients with higher TSAT values in various groups (i.e., LAH, HA and H) had a lower risk of death and composite events. These results are consistent with the results of previous studies. In the general population, both low (<24%) and high (>40%) TSAT ratios are significantly and independently associated with increased all-cause and cardiovascular-specific mortality [12]. Similarly, H.M. Koo et al. reported that both low (≤20%) and high (40%) TSAT levels in HD patients were associated with a higher risk for all-cause and cardiovascular mortality death [13]. From these reports and the results of our own study, we hypothesized that maintenance of higher TSAT levels via iron administration in patients with low TSAT level might contribute to the improvement of overall mortality and/or decrease the risk of adverse events in MHD patients. However, in this study, patient gender, age, comorbidities (e.g., diabetes), hCRP level, and β2-MG levels were identified as significant predictors of TSAT levels in MHD patients, whereas the dosage of intravenous iron or iron storage (e.g., serum ferritin levels) were not identified as such. Furthermore, there was no significant difference in TSAT levels between patients who did or did not receive intravenous iron for more than 3 months. From these results, we that normal or higher TSAT levels, which were associated with a lower risk of adverse events or death in MHD patients, were primarily regulated by clinical conditions in MHD patients and were not necessarily achieved via intravenous iron supplementation. On the other hand, as Fig 4 indicates, patients with absolute iron deficiency (both low ferritin and low TSAT levels) needed IV iron to maintain the target Hb or TSAT level. Previous guidelines associated with iron deficiency [4] have reported that, because of poor sensitivity and specificity in the detection of responsiveness to parenteral iron therapy in CKD patients, isolated TSAT levels are not recommended as an index for determining the initiation of iron administration. Therefore, it is possible that although low TSAT levels can predict anemia, adverse events and survival in MHD patients, iron administration based on low TSAT levels alone does not necessarily decrease the risk of adverse events or premature death.

### Iron utilization for erythropoiesis and adverse events in HMD patients

In this study, we also found that patients with high ferritin and low TSAT levels showed a significantly higher risk of CCVD and all-cause mortality. This result indicated that among MHD patients, patients with dysutalization of iron for erythropoiesis show a greater risk for adverse events or death. High serum ferritin with low TSAT levels may imply a condition of iron sequestration. Partial blockade of iron transport to erythroid marrow occurs in several clinical conditions, such as anemia of chronic disease [14]. Hepcidin exerts its iron-regulatory effects by binding to the transmembrane iron exporter ferroportin, causing cellular ferroportin internalization and degradation that subsequently decreases the iron available for heme/hemoglobin synthesis in erythroid cells [15]. Moreover, it has been reported that hepcidin may contribute to anemia in association with inflammation not only through effects on iron metabolism but also through inhibition of erythroid progenitor proliferation and survival [16]. Although we did not evaluate hepcidin levels in this study, we have previously reported that serum hepcidin levels in MHD patient are significantly higher than those of healthy volunteers, and serum hepcidin in these patients was closely associated with serum ferritin levels [17]. Moreover, elevated serum hepcidin levels in chronic kidney disease patients (not on dialysis) with <TSAT 20% and ferritin levels ≥40 ng/mL have been reported [18]. Therefore, it is possible that serum hepcidin levels in patients with dysutalization iron for erythropoiesis might be higher than in other patient groups. Recently, several studies have reported an association between overexpression of hepcidin and cardiovascular disease. Li JJ et al. showed that expression of hepcidin was closely associated with the upregulation of atherosclerotic plaque formation and plaque instability [19]. We also previously identified an association between higher serum hepcidin levels and increased pulse wave velocity (PWV), which indicates vascular stiffness [20]. Furthermore, an observational study performed on 405 MHD patients for 3 years reported that higher serum hepcidin levels were significantly associated with a higher risk for cardiovascular disease-related death [21]. Although hepcidin is not toxic itself, overexpression of hepcidin might cause iron sequestration in several cells and tissues or dysutilization of iron for erythropoiesis. From our results and previous studies, overexpression of hepcidin in patients with dysutilization of iron for erythropoiesis might cause higher risks of adverse events or premature death in these patients.

### Iron administration in patients with dysutalization of iron for erythropoiesis

In this study, we also found that patients with high TSAT levels and high ferritin levels had a significantly higher risk of death than other patient groups. The Drive study [7] reported that iron administration in patients with dysutilization of iron for erythropoiesis might attenuate the responsiveness to ESA and increase Hb levels. However, we have previously reported that, among patients with hyporesponsiveness to ESA and patients with higher ferritin level (>100 ng/mL), those treated with higher doses of intravenous iron showed a higher risk of composite events (e.g., CCVD, infection, hospitalization, and death) [18]. We recently demonstrated that a high risk of death and/or adverse events was associated with a consistently high ferritin level, large fluctuations in ferritin levels and high doses of intravenous iron [9] in MHD patients. Moreover, a nationwide Japanese registry-based cohort study also reported that the risk of all-cause death increased along with increased serum ferritin levels in MHD patients [22]. From the results of our study and previous studies, we hypothesize that the additional administration of iron in patients with dysutilization of iron for erythropoiesis increases iron stores and induces the overexpression of hepcidin, which is associated with a higher risk of adverse events and premature death in these patients.

## Conclusion

Patients with low TSAT levels had a significantly higher risk of CCVD and death. High TSAT levels were associated with the clinical and demographic background of MHD patients (including patient gender, age, inflammatory conditions, and comorbidities) but not with iron administration or iron storage. Patients with low ferritin (<100 ng/mL) and low TSAT (<20%) levels were not at a significantly increased risk of adverse events or death. On the other hand, patients with high ferritin (≥100 ng/mL) and low TSAT (<20%) levels, who were suspected as presenting dysutilization of iron for erythropoiesis had a higher risk of CCVD and death. From these results, the administration of iron should be approached with caution in patients who present with dysutilization of iron for erythropoiesis. Further prospective randomized control studies are needed to validate these findings and whether iron administration could increase TSAT for long-term period and improve the prognosis.

## Supporting information

S1 Data set (DOCX)

## References

[1] Locatelli F, Nissenson AR, Barrett BJ, Walker RG, Wheeler DC, Eckardt KU et al Clinical practice guidelines for anemia in chronic kidney disease: problems and solutions. A position statement from Kidney Disease: Improving Global Outcomes (KDIGO). Kidney Int. 2008;74(10):1237–40 10.1038/ki.2008.299 18596731

[2] 2015 Japanese Society for Dialysis Therapy: Guidelines for Renal Anemia in Chronic Kidney Disease. Renal Replacement Therapy 2017; 3: 36.

[3] Thomas DW, Hinchliffe RF, Briggs C, Macdougall IC, Littlewood T, Cavill I; British Committee for Standards in Haematology. Guideline for the laboratory diagnosis of functional iron deficiency. Br J Haematol. 2013 161(5):639–48. 10.1111/bjh.12311 23573815

[4] Elsayed ME, Sharif MU, Stack AG. Transferrin Saturation: A Body Iron Biomarker. Adv Clin Chem. 2016;75:71–97. 10.1016/bs.acc.2016.03.002 27346617

[5] Hashemi SM, Mashhadi MA, Mohammadi M, Ebrahimi M, Allahyari A. Absolute and Functional Iron Deficiency Anemia among Different Tumors in Cancer Patients in South Part of Iran, 2014. Int J Hematol Oncol Stem Cell Res. 2017 1;11(3):192–198. 28989585 PMC5625469

[6] Przybylowski P, Wasilewski G, Golabek K, Bachorzewska-Gajewska H, Dobrzycki S, Koc-Zorawska E et al Absolute and Functional Iron Deficiency Is a Common Finding in Patients With Heart Failure and After Heart Transplantation. Transplant Proc. 2016 48(1):173–6. 10.1016/j.transproceed.2015.12.023 26915864

[7] Kapoian T, O’Mara NB, Singh AK, Moran J, Rizkala AR, Geronemus R et al Ferric gluconate reduces epoetin requirements in hemodialysis patients with elevated ferritin. J Am Soc Nephrol. 2008 19(2):372–9. 10.1681/ASN.2007050606 18216316 PMC2396742

[8] Susantitaphong P, Alqahtani F, Jaber BL. Efficacy and safety of intravenous iron therapy for functional iron deficiency anemia in hemodialysis patients: a meta-analysis. Am J Nephrol. 2014;39(2):130–41. 10.1159/000358336 24513913

[9] Kuragano T, Matsumura O, Matsuda A, Hara T, Kiyomoto H, Murata T et al Association between hemoglobin variability, serum ferritin levels, and adverse events/mortality in maintenance hemodialysis patients. Kidney Int. 2014 86(4):845–54. 10.1038/ki.2014.114 24759150

[10] Wish JB. Assessing iron status: beyond serum ferritin and transferrin saturation. Clin J Am Soc Nephrol. 2006 1 Suppl 1:S4–8. 17699374 10.2215/CJN.01490506

[11] Tsubakihara Y, Nishi S, Akiba T, Hirakata H, Iseki K, Kubota M, et al 2008 Japanese Society for Dialysis Therapy: Guidelines for Renal Anemia in Chronic Kidney Disease. Ther Apher Dial. 2010;14(3):240–75. 10.1111/j.1744-9987.2010.00836.x 20609178

[12] Stack AG, Mutwali AI, Nguyen HT, Cronin CJ, Casserly LF, Ferguson J. Transferrin saturation ratio and risk of total and cardiovascular mortality in the general population. QJM. 2014 107(8):623–33. 10.1093/qjmed/hcu045 24599805 PMC4108849

[13] Koo HM, Kim CH, Doh FM, Lee MJ, Kim EJ, Han JH et al The relationship of initial transferrin saturation to cardiovascular parameters and outcomes in patients initiating dialysis. PLoS One. 2014 5;9(2):e87231 10.1371/journal.pone.0087231 24505281 PMC3914817

[14] Hashemi SM, Mashhadi MA, Mohammadi M, Ebrahimi M, Allahyari A. Absolute and Functional Iron Deficiency Anemia among Different Tumors in Cancer Patients in South Part of Iran, 2014. Int J Hematol Oncol Stem Cell Res. 2017 1;11(3):192–198. 28989585 PMC5625469

[15] Nakanishi T, Hasuike Y, Otaki Y, Kida A, Nonoguchi H, Kuragano T. Hepcidin: another culprit for complications in patients with chronic kidney disease? Nephrol Dial Transplant. 2011 26(10):3092–100. 10.1093/ndt/gfr410 21785039

[16] Dallalio G, Law E, Means T: Hepcidin inhibits in vitro erythroid colony formation at reduced erythropoietin. Blood 2006; 107: 2702–2704. 10.1182/blood-2005-07-2854 16332970 PMC1895381

[17] Kuragano T, Shimonaka Y, Kida A, Furuta M, Nanami M, Otaki Y et al Determinants of hepcidin in patients on maintenance hemodialysis: role of inflammation. Am J Nephrol. 2010;31(6):534–40. 10.1159/000312381 20484891

[18] Kuragano T, Kitamura K, Matsumura O, Matsuda A, Hara T, Kiyomoto H et al ESA Hyporesponsiveness Is Associated with Adverse Events in Maintenance Hemodialysis (MHD) Patients, But Not with Iron Storage. PLoS One. 2016 2;11(3):e0147328 10.1371/journal.pone.0147328 26933949 PMC4774978

[19] Li JJ, Meng X, Si HP, Zhang C, Lv HX, Zhao YX et al Hepcidin destabilizes atherosclerotic plaque via overactivating macrophages after erythrophagocytosis. Arterioscler Thromb Vasc Biol. 2012;32(5):1158–66. 10.1161/ATVBAHA.112.246108 22383698

[20] Kuragano T, Itoh K, Shimonaka Y, Kida A, Furuta M, Kitamura R et al Hepcidin as well as TNF-α are significant predictors of arterial stiffness in patients on maintenance hemodialysis. Nephrol Dial Transplant. 2011;26(8):2663–7. 10.1093/ndt/gfq760 21245128

[21] van der Weerd NC, Grooteman MP, Bots ML, van den Dorpel MA, den Hoedt CH, Mazairac AH. et al CONTRAST Investigators. Hepcidin-25 is related to cardiovascular events in chronic haemodialysis patients. Nephrol Dial Transplant. 2013;28(12):3062–71. 10.1093/ndt/gfs488 23147161

[22] Maruyama Y, Yokoyama K, Yokoo T, Shigematsu T, Iseki K, Tsubakihara Y. The Different Association between Serum Ferritin and Mortality in Hemodialysis and Peritoneal Dialysis Patients Using Japanese Nationwide Dialysis Registry. PLoS One. 2015 23;10(11):e0143430 10.1371/journal.pone.0143430 26599216 PMC4658129

