# Supplementary material for: Low transferrin saturation (TSAT) and high ferritin levels are significant predictors for cerebrovascular and cardiovascular disease and death in maintenance hemodialysis patients
Source: PLoS One. 2020 Sep 2;15(9):e0236277. doi: 10.1371/journal.pone.0236277 (PMC7467218; doi:10.1371/journal.pone.0236277)
Supplement: S1 Data set — (DOCX) [file pone.0236277.s001.docx]

1. The results of ferritin categories

Results of Linear regression analysis

| **descriptive statistics** | | | | |
| --- | --- | --- | --- | --- |
| Ferritin mean 100_200_300 | | mean | SD | Case |
| 100ng/mL under | Ferritin Ln | 3.7028 | .57060 | 496 |
|  | TSAT平均 | 24.7249 | 7.2406 | 496 |
| 100ng/mL over 200ng/mL under | Ferritin Ln | 4.9368 | .17837 | 168 |
|  | TSAT平均 | 28.5947 | 7.3429 | 168 |
| 200ng/mL over 300ng/mL under | Ferritin Ln | 5.4357 | .12201 | 32 |
|  | TSAT | 30.5081 | 10.1754 | 32 |
| 300ng/mL over | Ferritin Ln | 6.1594 | .36554 | 28 |
|  | TSAT | 36.3235 | 15.1447 | 28 |
| 100ng/mL over | Ferritin Ln | 5.1570 | .46057 | 228 |
|  | TSAT | 29.8124 | 9.3532 | 228 |

| Ferritin mean | | R | R2 | Adjust R2 | Sd |
| --- | --- | --- | --- | --- | --- |
|  |  |  |  |  |  |
| 100ng/mL under | 1 | .270^b^ | .073 | .071 | .55003 |
| 100ng/mL over 200ng/mL under | 1 | .129^b^ | .017 | .011 | .17741 |
| 200ng/mL over 300ng/mL under | 1 | .038^b^ | .001 | -.032 | .12394 |
| 300ng/ml over | 1 | .455^b^ | .207 | .176 | .33179 |
| 100ng/mL over | 1 | .336^b^ | .113 | .109 | .43472 |

| Ferritin mean 100_200_300 | | | Non adjust | | Adjust | t | P | 95.0% CI | |
| --- | --- | --- | --- | --- | --- | --- | --- | --- | --- |
|  |  |  | B | SD |  |  |  | lower | under |
| 100ng/mL under | 1 |  | 3.178 | .088 |  | 36.125 | .000 | 3.005 | 3.350 |
|  |  | TSAT | .021 | .003 | .270 | 6.222 | .000 | .015 | .028 |
| 100ng/mL over 200ng/mL under | 1 |  | 4.847 | .055 |  | 87.834 | .000 | 4.738 | 4.956 |
|  |  | TSAT | .003 | .002 | .129 | 1.677 | .095 | -.001 | .007 |
| 200ng/mL over 300ng/mL under | 1 |  | 5.450 | .070 |  | 77.581 | .000 | 5.306 | 5.593 |
|  |  | TSAT | .000 | .002 | -.038 | -.207 | .837 | -.005 | .004 |
| 300ng/mL over | 1 |  | 5.761 | .165 |  | 34.812 | .000 | 5.421 | 6.101 |
|  |  | TSAT mean | .011 | .004 | .455 | 2.602 | .015 | .002 | .020 |
| 100ng/mL over | 1 |  | 4.663 | .096 |  | 48.391 | .000 | 4.474 | 4.853 |
|  |  | TSAT mean | .017 | .003 | .336 | 5.366 | .000 | .010 | .023 |

Spearman

| Ferritin mean | | | Ferritin Ln mean |
| --- | --- | --- | --- |
| 100ng/mL under | TSAT mean | correlation coefficients | .259^**^ |
|  |  | P | .000 |
|  |  | n | 496 |
| 100ng/mL over200ng/mL under | TSAT mean | correlation coefficients | .137 |
|  |  | P | .078 |
|  |  | n | 168 |
| 200ng/mL over 300ng/mL under | TSAT mean | correlation coefficients | -.076 |
|  |  | P | .678 |
|  |  | n | 32 |
| 300ng/mL over | TSAT mean | correlation coefficients | .458^*^ |
|  |  | P | .014 |
|  |  | n | 28 |
| 100ng/mL over | TSAT mean | correlation coefficients | .177^**^ |
|  |  | P | .007 |
|  |  | n | 228 |

２）The determines of TSAT (LASSO analysis）。

Lambda= 0.006218239

|  | Independent | coefficient | Odd ratio |
| --- | --- | --- | --- |
| *Ｘ_1_* | CRP | -0.3381 | 0.7132 |
| *Ｘ_2_* | Ferritin | 0.8634 | 2.3713 |
| *Ｘ_3_* | β2MG | -0.0694 | 0.9330 |
| *Ｘ_4_* | DM | -0.5281 | 0.5897 |
| *Ｘ_5_* | CVD | -0.3260 | 0.7218 |
| *Ｘ_6_* | TP | -0.2882 | 0.7496 |
| *Ｘ_7_* | Hb | 0.1655 | 1.1799 |
| *Ｘ_8_* | Sex | -0.4028 | 0.6684 |
| *Ｘ_9_* | int_PTH | -0.1163 | 0.8902 |
| *Ｘ_10_* | Age | -0.0103 | 0.9898 |
| *Ｘ_11_* | ESA dose | 0.0001 | 1.0001 |
| *Ｘ_12_* | BMI | -0.0114 | 0.9887 |
|  | intercept | 0.7166 |  |

|  | | Result | | |
| --- | --- | --- | --- | --- |
|  |  | TSAT20% over | TSAT20% under | total |
| presupposition | TSAT20% under | 332 | 79 | 411 |
|  | TSAT20% over | 9 | 17 | 26 |
|  | Total | 341 | 96 | 437 |

|  |  |  | 95%C.I. |
| --- | --- | --- | --- |
| Prevalence |  | 0.780 | 0.739 - 0.818 |
| sensitivity |  | 0.974 | 0.950 - 0.988 |
| specificity |  | 0.177 | 0.107 - 0.268 |
| positive predictive value | | 0.808 | 0.766 - 0.845 |
| negative predictive value | | 0.654 | 0.443 - 0.828 |
| accuracy |  | 0.799 | 0.758 - 0.835 |
| false positive rate |  | 0.823 | 0.732 - 0.893 |
| false negative rate |  | 0.026 | 0.012 - 0.05 |

The relationship between TSAT and iron administration

|  |  | 0M | 3M | 6M | 9M | 12M | 15M | 18M |
| --- | --- | --- | --- | --- | --- | --- | --- | --- |
| TSAT20% under | N | 143 | 141 | 135 | 128 | 123 | 118 | 112 |
|  | mean | 11.19 | 14.89 | 15.37 | 12.70 | 11.38 | 10.59 | 21.65 |
|  | SD | 31.91 | 32.73 | 34.69 | 34.37 | 32.53 | 26.64 | 37.89 |
|  | min | 0.0 | 0.0 | 0.0 | 0.0 | 0.0 | 0.0 | 0.0 |
|  | max | 150.0 | 125.0 | 125.0 | 150.0 | 150.0 | 125.0 | 125.0 |
|  | median | 0.0 | 0.0 | 0.0 | 0.0 | 0.0 | 0.0 | 0.0 |
|  | Percentile 25 | 0.0 | 0.0 | 0.0 | 0.0 | 0.0 | 0.0 | 0.0 |
|  | Percentile 75 | 0.0 | 25.0 | 25.0 | 0.0 | 0.0 | 0.0 | 25.0 |
| TSAT20% over | N | 582 | 579 | 571 | 560 | 541 | 524 | 511 |
|  | mean | 10.65 | 12.22 | 11.78 | 9.96 | 9.75 | 10.21 | 11.45 |
|  | SD | 27.56 | 26.81 | 27.07 | 26.92 | 25.06 | 25.93 | 26.50 |
|  | min | 0.0 | 0.0 | 0.0 | 0.0 | 0.0 | 0.0 | 0.0 |
|  | max | 125.0 | 150.0 | 125.0 | 150.0 | 125.0 | 150.0 | 150.0 |
|  | median | 0.0 | 0.0 | 0.0 | 0.0 | 0.0 | 0.0 | 0.0 |
|  | Percentile 25 | 0.0 | 0.0 | 0.0 | 0.0 | 0.0 | 0.0 | 0.0 |
|  | Percentile 75 | 0.0 | 25.0 | 25.0 | 0.0 | 0.0 | 0.0 | 25.0 |
|  |  |  |  |  |  |  |  |  |
|  |  | 21M | 24M | 27M | 30M | 33M | 36M |  |
| TSAT20% under | N | 110 | 102 | 95 | 84 | 78 | 64 |  |
|  | mean | 11.59 | 18.14 | 11.84 | 14.29 | 18.91 | 14.06 |  |
|  | SD | 28.87 | 35.56 | 25.22 | 27.24 | 33.51 | 24.35 |  |
|  | min | 0.0 | 0.0 | 0.0 | 0.0 | 0.0 | 0.0 |  |
|  | max | 125.0 | 125.0 | 125.0 | 125.0 | 125.0 | 125.0 |  |
|  | median | 0.0 | 0.0 | 0.0 | 0.0 | 0.0 | 0.0 |  |
|  | Percentile 25 | 0.0 | 0.0 | 0.0 | 0.0 | 0.0 | 0.0 |  |
|  | Percentile 75 | 0.0 | 25.0 | 25.0 | 25.0 | 25.0 | 25.0 |  |
| TSAT20% over | N | 496 | 481 | 471 | 436 | 431 | 334 |  |
|  | mean | 11.19 | 13.20 | 13.11 | 15.77 | 15.37 | 12.13 |  |
|  | SD | 26.80 | 27.48 | 28.42 | 29.01 | 27.86 | 26.83 |  |
|  | min | 0.0 | 0.0 | 0.0 | 0.0 | 0.0 | 0.0 |  |
|  | max | 125.0 | 125.0 | 125.0 | 150.0 | 150.0 | 150.0 |  |
|  | median | 0.0 | 0.0 | 0.0 | 0.0 | 0.0 | 0.0 |  |
|  | Percentile 25 | 0.0 | 0.0 | 0.0 | 0.0 | 0.0 | 0.0 |  |
|  | Percentile 75 | 12.5 | 25.0 | 25.0 | 25.0 | 25.0 | 25.0 |  |

|  | | TSAT | |
| --- | --- | --- | --- |
|  |  | TSAT20% under | TSAT20% over |
| Iron administaration | N | 144 | 581 |
|  | mean | 14.067 | 11.731 |
|  | SD | 21.529 | 15.618 |
|  | min | 0.00 | 0.00 |
|  | max | 140.00 | 125.00 |
|  | median | 8.01 | 8.33 |
|  | Percentile 25 | 1.92 | 2.08 |
|  | Percentile 75 | 15.50 | 13.46 |

Mann-Whitney’s U-test

| **Rank** | | | | |
| --- | --- | --- | --- | --- |
| TSAT mean | | Frequency | rank | Rank sam |
| Iron administaration | TSAT20% under | 144 | 365.91 | 52691.00 |
|  | TSAT20% over | 581 | 362.28 | 210484.00 |
|  | Total | 725 |  |  |

| **Official approval estimated quantity** | |
| --- | --- |
|  | Iron administaration |
| Mann-Whitney U | 41413.000 |
| Wilcoxon W | 210484.000 |
| Z | -.187 |
| Asymptotic intentionality probability (both sides) | .852 |

Iron administration and mean TSAT

Description statistic (non-adjustment simple tabulation)

|  |  | 0M | 3M | 6M | 9M | 12M | 15M | 18M |
| --- | --- | --- | --- | --- | --- | --- | --- | --- |
| Iron non | ｎ | 135 | 118 | 110 | 102 | 89 | 79 | 74 |
|  | Mean | 25.94 | 23.30 | 26.02 | 28.27 | 28.29 | 27.05 | 24.32 |
|  | SD | 11.14 | 9.55 | 12.61 | 15.30 | 25.08 | 16.90 | 10.28 |
| Iron yes | ｎ | 587 | 575 | 545 | 565 | 549 | 529 | 516 |
|  | Mean | 26.76 | 26.03 | 27.02 | 30.04 | 26.24 | 25.20 | 26.10 |
|  | SD | 11.75 | 11.62 | 14.93 | 15.73 | 11.93 | 14.92 | 18.19 |
|  |  |  |  |  |  |  |  |  |
|  |  | 21M | 24M | 27M | 30M | 33M | 36M |  |
| Iron non | ｎ | 71 | 69 | 62 | 53 | 57 | 45 |  |
|  | Mean | 27.70 | 30.02 | 24.94 | 26.85 | 28.08 | 29.23 |  |
|  | SD | 15.71 | 16.45 | 11.53 | 15.02 | 14.51 | 17.40 |  |
| Iron yes | ｎ | 502 | 496 | 484 | 446 | 439 | 337 |  |
|  | Mean | 25.59 | 26.33 | 24.12 | 25.34 | 26.24 | 25.47 |  |
|  | SD | 11.46 | 21.76 | 10.58 | 13.92 | 11.96 | 14.25 |  |

The relationship between age and TSAT ferritin

|  | | Ferritin 100ng/mL under・TSAT20% under | Ferritin 100ng/mL under・TSAT20% over | Ferritin  100ng/mL over・TSAT20% under | Ferritin 100ng/mL over・TSAT20% over |
| --- | --- | --- | --- | --- | --- |
| Age | N | 118 | 377 | 25 | 203 |
|  | mean | 61.65 | 60.58 | 63.56 | 63.48 |
|  | SD | 9.36 | 10.01 | 8.58 | 9.51 |
|  | min | 34.0 | 26.0 | 38.0 | 33.0 |
|  | max | 83.0 | 80.0 | 76.0 | 86.0 |
|  | median | 62.0 | 62.0 | 65.0 | 66.0 |
|  | Percentile 25 | 57.0 | 55.0 | 59.0 | 59.0 |
|  | Percentile 75 | 69.0 | 68.0 | 69.0 | 70.0 |
| P-value vs ferritin 100ng/mL under・TSAT20% under | | Reference | 0.720 | 0.810 | 0.367 |
| P-value vs ferritin 100ng/mL under・TSAT20% over | | 0.720 | Reference | 0.446 | 0.004 |
| P-value vs ferritin 100ng/mL over・TSAT20% under | | 0.810 | 0.446 | Reference | 1.000 |
| P-value vs ferritin 100ng/mL over・TSAT20% over | | 0.367 | 0.004 | 1.000 | Reference |
| Tukey HSD test | | | | | |

The relationship between albumin and ferritin or TSAT

|  | | Ferritin 100ng/mL under・TSAT20% under | | | | Ferritin 100ng/mL under・TSAT20% over | Ferritin  100ng/mL over・TSAT20% under | Ferritin 100ng/mL over・TSAT20% over |
| --- | --- | --- | --- | --- | --- | --- | --- | --- |
| Alb平 | N | 118 | | | | 376 | 23 | 202 |
|  | mean | 3.73 | | | | 3.75 | 3.79 | 3.74 |
|  | SD | 0.28 | | | | 0.28 | 0.36 | 0.27 |
|  | min | 3.0 | | | | 2.7 | 3.0 | 3.0 |
|  | max | 4.4 | | | | 4.4 | 4.3 | 4.5 |
|  | median | 3.8 | | | | 3.8 | 3.8 | 3.7 |
|  | Percentile 25 | 3.6 | | | | 3.6 | 3.6 | 3.6 |
|  | Percentile 75 | 3.9 | | | | 3.9 | 4.0 | 3.9 |
| P-value vs ferritin 100ng/mL under・TSAT20% under | | 0.852 | 0.814 | 0.995 |  |  |  |  |
| P-value vs ferritin 100ng/mL under・TSAT20% over | | Reference | 0.949 | 0.917 |  |  |  |  |
| P-value vs ferritin 100ng/mL over・TSAT20% under | | 0.949 | Reference | 0.862 |  |  |  |  |
| P-value vs ferritin 100ng/mL over・TSAT20% over | | 0.917 | 0.862 | Reference |  |  |  |  |
| Tukey HSD test | | | | | | | | |

The relationship between β2MG and ferritin or TSAT

|  | | Ferritin 100ng/mL under・TSAT20% under | Ferritin 100ng/mL under・TSAT20% over | Ferritin  100ng/mL over・TSAT20% under | Ferritin 100ng/mL over・TSAT20% over |
| --- | --- | --- | --- | --- | --- |
| Beta2MG | N | 111 | 364 | 20 | 186 |
|  | mean | 28.41 | 26.37 | 26.30 | 26.31 |
|  | SD | 5.87 | 5.51 | 7.19 | 5.84 |
|  | min | 13.6 | 6.0 | 1.7 | 1.5 |
|  | max | 48.1 | 52.6 | 36.1 | 47.4 |
|  | median | 27.9 | 26.0 | 26.3 | 26.6 |
|  | Percentile 25 | 24.6 | 23.3 | 24.0 | 23.1 |
|  | Percentile 75 | 31.4 | 29.3 | 31.4 | 29.1 |
| P-value vs ferritin 100ng/mL under・TSAT20% under | | Reference | 0.006 | 0.423 | 0.012 |
| P-value vs ferritin 100ng/mL under・TSAT20% over | | 0.006 | Reference | 1.000 | 0.999 |
| P-value vs ferritin 100ng/mL over・TSAT20% under | | 0.423 | 1.000 | Reference | 1.000 |
| P-value vs ferritin 100ng/mL over・TSAT20% over | | 0.012 | 0.999 | 1.000 | Reference |
| Tukey HSD test | | | | | |

The relationship between CRP and ferritin or TSAT

|  | | Ferritin 100ng/mL under・TSAT20% under | Ferritin 100ng/mL under・TSAT20% over | Ferritin  100ng/mL over・TSAT20% under | Ferritin 100ng/mL over・TSAT20% over |
| --- | --- | --- | --- | --- | --- |
| CRP_ | N | 115 | 371 | 21 | 194 |
|  | mean | -2.15 | -3.05 | -2.77 | -2.77 |
|  | SD | 1.51 | 1.65 | 2.64 | 1.59 |
|  | min | -8.6 | -9.2 | -9.2 | -9.2 |
|  | max | 1.0 | .5 | .4 | .8 |
|  | median | -2.1 | -2.9 | -2.2 | -2.7 |
|  | Percentile 25 | -2.8 | -3.9 | -2.9 | -3.5 |
|  | Percentile 75 | -1.2 | -1.9 | -1.6 | -1.7 |
| P-value vs ferritin 100ng/mL under・TSAT20% under | | Reference | 0.000 | 0.724 | 0.005 |
| P-value vs ferritin 100ng/mL under・TSAT20% over | | 0.000 | Reference | 0.964 | 0.205 |
| P-value vs ferritin 100ng/mL over・TSAT20% under | | 0.724 | 0.964 | Reference | 1.000 |
| P-value vs ferritin 100ng/mL over・TSAT20% over | | 0.005 | 0.205 | 1.000 | Reference |
| Games-Howell test | | | | | |

## The relationship between Cr and ferritin or TSAT

|  | | Ferritin 100ng/mL under・TSAT20% under | Ferritin 100ng/mL under・TSAT20% over | Ferritin  100ng/mL over・TSAT20% under | Ferritin 100ng/mL over・TSAT20% over |
| --- | --- | --- | --- | --- | --- |
| Cr | N | 119 | 377 | 25 | 203 |
|  | mean | 11.23 | 11.27 | 10.92 | 10.46 |
|  | SD | 2.40 | 2.23 | 2.48 | 2.22 |
|  | min | 5.0 | 2.1 | 6.8 | 3.0 |
|  | max | 16.6 | 19.0 | 15.5 | 16.9 |
|  | median | 11.3 | 11.2 | 10.9 | 10.4 |
|  | Percentile 25 | 9.4 | 9.9 | 9.3 | 9.0 |
|  | Percentile 75 | 13.1 | 12.9 | 12.6 | 11.7 |
| P-value vs ferritin 100ng/mL under・TSAT20% under | | Reference | 0.998 | 0.925 | 0.017 |
| P-value vs ferritin 100ng/mL under・TSAT20% over | | 0.998 | Reference | 0.875 | 0.000 |
| P-value vs ferritin 100ng/mL over・TSAT20% under | | 0.925 | 0.875 | Reference | 0.768 |
| P-value vs ferritin 100ng/mL over・TSAT20% over | | 0.017 | 0.000 | 0.768 | Reference |
| Tukey HSD test | | | | | |

The relationship between TSAT ferritin categories

|  | | Ferritin x TSAT | | | | | | | |
| --- | --- | --- | --- | --- | --- | --- | --- | --- | --- |
|  |  | ferritin100ng/mL under・TSAT20% under | | Ferritin 100ng/mL under・TSAT20% over | | Ferritin 100ng/mL over・TSAT20% under | | Ferritin 100ng/mL over・TSAT20% over | |
|  |  | ｎ | ％ | ｎ | ％ | ｎ | ％ | ｎ | ％ |
| CVD | yes | 2015 | 98.9% | 2807 | 99.5% | 461 | 96.8% | 2357 | 98.3% |
|  | no | 22 | 1.1% | 15 | .5% | 15 | 3.2% | 40 | 1.7% |
|  | total | 2037 | 100.0% | 2822 | 100.0% | 476 | 100.0% | 2397 | 100.0% |
| infection | yes | 2028 | 99.6% | 2811 | 99.6% | 475 | 99.8% | 2393 | 99.8% |
|  | no | 9 | .4% | 11 | .4% | 1 | .2% | 4 | .2% |
|  | total | 2037 | 100.0% | 2822 | 100.0% | 476 | 100.0% | 2397 | 100.0% |
| Hospitalization | yes | 1926 | 94.6% | 2733 | 96.8% | 439 | 92.2% | 2304 | 96.1% |
|  | no | 111 | 5.4% | 89 | 3.2% | 37 | 7.8% | 93 | 3.9% |
|  | total | 2037 | 100.0% | 2822 | 100.0% | 476 | 100.0% | 2397 | 100.0% |
| Death | yes | 2022 | 99.3% | 2813 | 99.7% | 463 | 97.3% | 2378 | 99.2% |
|  | no | 15 | .7% | 9 | .3% | 13 | 2.7% | 19 | .8% |
|  | total | 2037 | 100.0% | 2822 | 100.0% | 476 | 100.0% | 2397 | 100.0% |
| Composite | yes | 1916 | 94.1% | 2724 | 96.5% | 431 | 90.5% | 2280 | 95.1% |
|  | no | 121 | 5.9% | 98 | 3.5% | 45 | 9.5% | 117 | 4.9% |
|  | total | 2037 | 100.0% | 2822 | 100.0% | 476 | 100.0% | 2397 | 100.0% |
